# COVID-19 crisis interlinkage with past pandemics and their effects on food security

**DOI:** 10.1186/s12992-023-00952-7

**Published:** 2023-07-31

**Authors:** Hynek Roubík, Michal Lošťák, Chama Theodore Ketuama, Jana Soukupová, Petr Procházka, Adam Hruška, Josef Hakl, Lukáš Pacek, Petr Karlík, Lucie Kocmánková Menšíková, Vladimíra Jurasová, Charles Amarachi Ogbu, Michal Hejcman

**Affiliations:** 1 Faculty of Tropical AgriSciences, Czech University of Life Sciences Prague, Kamýcká 129, 165 00 Prague, Czech Republic; 2 Faculty of Economics and Management, Czech University of Life Sciences Prague, Kamýcká 129, 165 00 Prague, Czech Republic; 3 Faculty of Environmental Sciences, Czech University of Life Sciences Prague, Kamýcká 129, 165 00 Prague, Czech Republic; 4 Faculty of Agrobiology, Food and Natural Resources, Czech University of Life Sciences Prague, Kamýcká 129, 165 00 Prague, Czech Republic; 5 Faculty of Forestry and Wood Sciences, Czech University of Life Sciences Prague, Kamýcká 129, 165 00 Prague, Czech Republic

**Keywords:** Covid-19, Pandemic, Food security, Food system, Resilience, Response, Policy

## Abstract

**Background:**

Pandemics as health and humanitarian crises have exerted traceable impacts on food security. Almost all past and current pandemics have created a food crisis that affects a share of the global population and threaten global food security. With the more frequent outbreaks of emerging and re-emerging diseases or pandemics, this paper looks at the various types of impacts from the current coronavirus crisis and past pandemics to identify their major impact on food security.

**Scope:**

To this effect, key strategies that could be put in place to ensure the efficient resilience of food systems before, during, and after the pandemics to mitigate the negative impact of the pandemics on global food security are recommended. The most recent effects of the current coronavirus crisis have been disruptions in the flow of farm labourers and inefficient farm operations leading to postharvest food losses.

**Key findings and conclusions:**

Modification of diets between social groups has also been observed. Future response orientations to prevent and mitigate the effects of pandemics on food security will consider pro-active and adapted policy, program, and institutional actions towards the systemic development of global food systems as an interconnected network.

## Highlights

• Before the 20^th^century hunger and illnesses might be traced to climatic changes.

• The catastrophes in human history elicit various reactions – defence, reflection.

• In the 19^th^century, lower quality of food further exacerbated the effects of famines.

• Skills required for effectivemanagement of the flow of goods and services are in high demand.

• Alternative food resources exist during pandemic and food shortage.

• Strategizing to ensure food access and self-sufficiency during a pandemic is possible.

## Introduction

The coronavirus disease (COVID-19) is an easily transmissible disease caused by the novel coronavirus SARS-CoV-2 [63]. This disease was identified in December 2019 in China and was afterwards declared as a pandemic by WHO in March 2020 [109]. Due to its spread COVID-19 has impacted health systems and the economy around the globe [76] and will potentially have severe secondary consequences in many fields [62, 106].

As highlighted by Peters et al. [76], global disease outbreaks and pandemics have been increasing exponentially over the last 40 years. There are numerous discussions about the reasons for this rapid growth, but among the most cited ones is the growth of the human population [40] linked with the destabilization of environments and ecosystems as well as linkage through globalization [2]. One of the key issues is human actions have largely affected animals in terms of both land use and climate change (as some animals functioning as disease vectors are forced to migrate more radically. Therefore, there is emerging need to re-strategize food production value chains with certain epidemics being linked to large scale farming (such as avian influenza-including the 2006 H5N1 epidemic,and swine influenza-including the 2009 H1N1 pandemic.

Furthermore, the results of a various forms of social distancing over the COVID-19 period (to fight the health crisis)have put millions of businesses, workers, and farmers at risk. Especially the “poorest of the poor” are placed at biggest threat [71].

As a pandemic is primarily geographic, it groups multiple, distinct types of individual and public health threats, all of which have their severity, frequency, and other disease characteristics. Pandemics have been known to cause sudden, widespread morbidity and mortality as well as social, political, and economic disruption [86]. EU countries like other parts of the world have been affected by several notable pandemics, including the Black Death, Spanish flu, human immunodeficiency virus/acquired immune deficiency syndrome (HIV/AIDS), and currently, the novel coronavirus disease, denoted COVID-19. Proper international cooperation helps in achieving the best global response needed to reduce the effects of the pandemics.

Especially because of the continuous rising level of risks and the more frequent outbreaks of emerging and re-emerging diseases that pose a threat to the human population, we need to consider impacts on agriculture and food security [26, 76]. If not, the destabilization of systems will be high, even if pandemics are not necessarily severe. The core of the current threat to food security stems from the combination of disrupted logistics as seen in case of COVID-19 [53] and the lack of available seasonal and low-skilled workforce for the agricultural production itself [9]. While the aforementioned animal disease outbreaks pose a wide threat, the experience with H5N1 and N1H1 enhanced the resilience of the industry [93]. Even though the contemporarily emergent threats remain a rather mild threat to the globalised food supply, both the regionality as well as relative short scale of the effects of the pandemic highlight a potential for greater future risks.

Therefore, the present paper focuses on highlighting the food security implications of the current COVID-19 crisis within the global context, while simultaneously reflecting on past disasters and pandemics, and their effects on food security of the population. Furthermore, the paper compares the empirical evidence of historical pandemics with the current COVID-19 pandemic, aiming to uncover the links between pandemics and food insecurity. Finally, the study highlights critical strategies taken in different regions and countries and presents the respective preventive roads aiming towards minimising the potential impacts and risks as well as approaches with the potential of being the ways “out of the crisis”. To meet this aim, this paper is divided into various sections. First, a special attention to pandemics and natural disasters in the twentieth century and prior pointing to their socioeconomic impacts. Then, the risks associated with these events around the globe are highlighted. Followed by the influence of COVID-19 on food production in different continents. In the latter sections, approaches applied to ensure food security as well as food and nutritional alternatives embraced during these crises are discussed.

### Historical overview of the effects of pandemics and natural disasters

#### Before the twentieth century

**Fig. 1:**
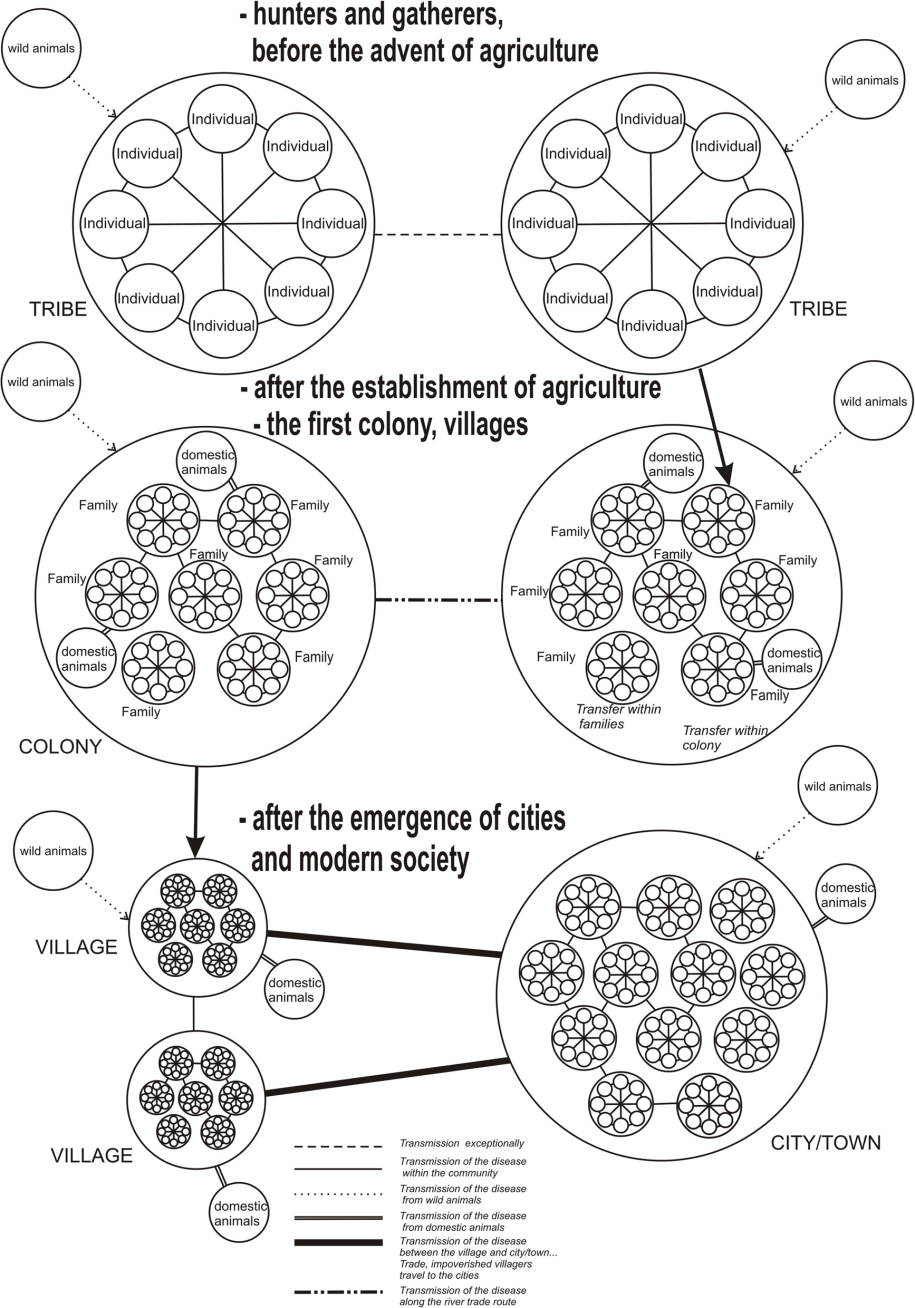
Visualization of pandemics spreads throughout the time (Authors original figure)

In medieval times, the word Black Death (plague) was used to denote all diseases with a high mortality rate. In terms of the prehistoric period, hunters and gatherers operated in vast territories, lacking prolonged contact between communities. That is why the probability of infectious disease transmission was low; it was limited to one tribe or kin [68, 92]. The first recorded forms of protection against the spread of disease can be dated to the tribal era– the other groups of hunters in the surroundings close to the infected community were usually warned to leave the place of infection. The neolithic revolution driven growth of the population related to the easier access to food provided by agriculture eroded such natural barriers [105]. The success in tilling the land and breading animals resulted in the establishment of permanent dwelling places in villages, towns, or cities and labour division. A large concentration of people in one place resulted in close contacts of people and accumulating wastes. This contributed to the spread of infectious diseases [36]. Trade was also another mean by which diseases were spread (Fig. 1). Original walking paths have changed into the tracks used by caravans with horses. Longer distances are operated by ships using rivers or seas. The sources of infections were also found inside farming communities. While domesticating the animals, people came in closer contact with the originators of diseases that might be transmitted by animals (Fig. 1). This is how influenza (poultry and pigs), smallpox and tuberculosis (cattle) were spread [35].

Rare documents from Central Europe, such as Chronica Boemorum recorded a very strong fear of the problems generated by crop failure [25]. The reason for such problems was believed to be caused by evil spells or the wrath of God. That is why people observe natural omens such as aurora (“blood on the sky”) or the invasions of grasshoppers (similar invasions were common in Europe until 1839) and very often the comets. Modern time uses science and rationality to explain issues causing bad yields or natural phenomena generating fear. We have the experience that strong frosts, drought, or floods might damage the yields even in the years when climatic conditions are relatively normal. Hunger and illness might not necessarily be the result of climate fluctuation for several years or due to local climatic effects [67]. Although the historic fear of crop failure is currently mostly mitigated by the aforementioned scientific perspective, the deep apprehension of hunger drives the contemporary populations into buying frenzy in times of crisis [54]. Therefore, the irrational approaches to individuals perceived food security led to a similar results for communities and regions as the historical examples.

When studying rare historical documents, we must be aware of bias researchers, the selectivity of human memory, and the story-telling nature of the documents. Despite these limitations, historical documents are very valuable for illustrating how the authors (with their peers) viewed hunger or epidemics in the context of natural processes or wars. This paper will present some of them with the goal of highlighting the relation between pandemics and food insecurity. Especially those, which reflect extraordinary cases in Europe (some of those targeted to the historical territory of today’s Czech Republic (CZ)).

The year 1282 started with an exceptionally harsh winter. The annals of the Czech chronicles [81] point out that “when the poor were refused entry to buildings in the city of Prague, at night they would hide in manure and lay on the city streets”. The spring started by Black Death (plague), the city was overcrowded, there were not enough burial grounds. Prague dug eight shafts for the dead, whose number amounted to thousands. The records suggest the link between an unfavourable year of 1281 and increased migration from rural areas to the city. The resulting overcrowding, lack of hygiene and spread of pathogens resulted in the aforementioned loss of lives.

At the beginning of the fourteenth century, a crisis broke out throughout Europe: Long-lasting rains and huge flooding in Silesia, Poland, and Bohemia caused great poverty and famine [51]. These events caused people in some places behaved like wild animals—they attacked each other, were throttling or eating each other. Such terrible famine lasted for three consecutive years. The historic notes suggest that the situation got worst in the year 1315. [80]. The lack of imported food to the affected areas followed the above mentioned pattern of distribution driven food insecurity, similar to the pandemic times.

The harvest in 1439 was ordinary but the end of this year was signified by a plague. Daniel Adam, duke of Veleslavín noted in his memoirs: “*A comet of the deathly pale colour showed up on the sky, it meant fear and death … because later on during this year in the days close to St. Laurent day, Black Death started in Bohemia and it lasted 13 weeks. In Prague, they buried one day hundred people and altogether 11,000 people died*”. The memories of the municipality of Plotiště nad Labem point out: “*who was poisoned by Black Death he slept three days and three nights and when he woke up, he immediately continued to the dead*” [79]. These were times in which there were no prior references to assist in the control and mitigation of the disease. The activities in affected societies were limited to certain provisions for dying people and when dead they were just buried.

The seventeenth-century provides reports of the food trades during the period of hunger. As Mikuláš Dačický describes the trade was accompanied by speculations. “*costs were high and were everywhere; especially in grains, some surrounding countries indicated their lack which caused hunger. For this reason, large quantities of grain that were stored by monetary misers waiting for even higher prices were exported*” [16], these events happened in 1617. In 1662 Antonín Strnad a Josef Stepling [99] wrote about the distribution of grain: “*The dryness of last year continued with extreme exorbitance. Armies plundered the settlements because of the hunger. Regions of Žatec, Litoměřice, and Plzeň assisted through grains*.”

Under the Austro-Hungarian monarchy, the food was already being distributed to localities heavily impacted by hunger. Ondřej Lukavský [90] writes in his memoirs: “*As for what did happen, because the last year 1746 already showed low harvest and extensive hunger and lack of bread, people consumed whatever plants because they had to eat. Since prices are too great and most of the food in Bohemia is unavailable but must be imported from Hungary or Moravia.*”

The second half of the eighteenth century is typified by several rainy years with a bad harvest. It is the time of “small pluvial” [104]. The situation in Czechia was not good, but, even under such circumstances, the food was taken away from the country. “*Last year (1796) although a small amount of wheat was here, all wheat was sold abroad to Bavaria because in Bavaria the damages were greater than in Bohemia. It resulted in such hunger that many people had no bread for almost the whole year and if there were no potatoes, which were eaten during this time, the people would die*” Hostaš [50].

Unfavourable weather and hunger are reflected in the book of chronicles. “The hunger was extreme, so people ate nettles or even soil. It resulted in diseases” [56]. “Due to the bad harvest of grain, there was extreme exorbitance. Mostly in mountains, people ate bran (middlings) and a sort of grass called saltbush (orache). Many beggars walked across villages asking for a small piece of bread” [13].

Periods of food shortage in Central Europe also continued in the nineteenth century. However, famine affecting large areas did not occur. This is due to the existence of certain food assistance among the countries within Europe in these times.

Generally, the empirical evidence suggests a continual stabilization of the food security within Europe in spite of a rising population as well as a number of existing threats. In terms of the threats themselves the records highlight a similar pattern of disruption of production followed by an uneven distribution of goods resulting in severe lack of available food. While majority of shortages were not caused by pandemics, all the mentioned cases culminated with the disease driven mortality resulting in major loss of lives.

### Look into the twentieth century

World War I at the beginning of the twentieth century reinforced food shortage and hunger. The cause of the food shortage was not climatic conditions (which were handled by the distribution of food) but the flow of the food to battlefronts [89] as well as the existing naval blockades [94]. The food market continuously eroded,food vouchers were introduced. Money lost value, deception and usury bloomed. At the end of World War I, food was of low quality, substitutes were used, and often insufficient even for ration [61]. In 1918, at the very end of World War I, the largest modern pandemics – Spanish flu exploded. The global estimations of death account for about 50 million people [95, 100].

It is most likely that the Spanish flu originated in one or all of several centres. In 1916 in the Chinese province of Shanxi a respiratory disease with rather mild symptoms emerged. Although China disassociated itself from World War I, it committed to supplying the so-called Chinese labour corps, who served in non-combat roles such as trench digging and railway repairs [55]. Chinese working teams travelled by ships in terrifying conditions to France, Belgium, and Russia. Most of the men were medically scrutinized but the medical examination targeted mostly tropical diseases. It might be the first centre of influenza [95].

The second centre of infection might be in Europe, in Étaples, one of the British medical and army complexes located at the river of Somma, where the soldiers and patients present were in close contact with wild birds. Currently, the river of Somma is a large nesting ground of various species and investigation centre of bird flu. In the period 1916–1917, the aforementioned medical complex records indicate the disease described by local doctors as festering bronchitis with symptoms similar to Spanish flu.

A mutation of the flu might have taken place and the strain became highly virulent [95, 102].

The third probable place for backtracking the origin of the Spanish flu was the U.S. state of Kansas. Local farmers indicated many deaths in 1917 because of pneumonia. The farmers were in close contact with poultry and swine. Close to the infected farmers was army camp, Funston. The soldiers located there exhibited similar symptoms several weeks later. Arguably, this might have been the site of the first infection [95]. While no-one place of origin can be fully verified as ground zero, the possibility of independent source regions serves as a potential explanation for the phenomena. The extraordinary mobility of the pandemics was conditioned by its virulence and by infected war soldiers who returned to their homes. During the war, the soldiers were exposed to unfavourable conditions, cold, humidity, hunger, and stress which weakened the immune system of the soldiers. Consequently, being closely stuffed in trenches and barracks the influenza was easy to spread among them [27]. The impacts of the pandemics were disastrous. The people who were at a productive age were dying. Losses were counted in all sectors of the economy, inoperative services, factories closed down, and doctors absent in clinics [41, 95, 101]. Entire families died in rural areas. Cities were at the brink of total infrastructural collapse as facilities were barely able to handle the burial of corpses. A continuous shortage of food, coal, and medicine was evident. The pandemics were counted in three waves, the second, typified by the highest mortality. Finally, in 1920 the Spanish flu was fading away [97].

The cases document that when a crisis caused by natural, warlike or other events happen, the skills of all of those who manage established flow of goods and services typical of modern societies, were not enough to maintain the existing needs of the population. In the nearby future, stability cannot be expected, but the sequences of radical changes and immediate oscillations in the triangle formed by people, food resources, and diseases are evident [68].

While the link between food insecurity and the Spanish influenza remains questionable, the emergence of the pandemic in the aftermath of a major food security crisis resembles the aforementioned pre-modern events. In terms of human perspective the catastrophes in history elicit various reactions – either defence and/or reflection. However, in some communities seemingly outdated atavisms emerge – mostly “combatting for food”,—group aversions and hostility. That is why we can observe pogroms targeting Jewish communities or witch-hunts. Also, in recent history, extraordinary riots and murders happened (Tokyo – earthquake in 1923, thousands of Koreans died). Sometimes certain social norms or social structures hinder the handling of catastrophes. Once they are over, the societies develop re-emerging initiatives; they use the experience and try to learn from what happened. In the past, people encountered disasters more often, they had more experience with them since they lived all their lives with such burdens. Nowadays, societies operate under the vision of being able to eliminate whatever catastrophe. Such an approach might be dangerous for all.

### Establishment of the empirical link between pandemics and food insecurity

The empirical evidence in chapter 2 suggests a link between food insecurity and pandemics on basis of both medieval and twentieth century pandemics. The connections stems from a feedback loop in which the initial state of food insecurity weakens the population, thus increasing the severity of a disease resulting in further disruption of food supply. As DeWitte & Wood [21] point out, the mortality of the Black death corelated with pre-existing health conditions which were heavily affected by individuals’ nutrition. Similar results in enhanced spread of infectious diseases due to poor nutrition can be traced in the cases of plague waves across rest of Europe as well as in case of the Spanish influenza at the final stages of the First World War.

While the varying source of food crises predating the aforementioned pandemics ranges from the change of climate [78] to massive conflicts [95], the underlying elements include urbanization, population growth and economic inequality. As Pribyl [78] suggests, the initial wave of Black death followed a climate driven food production disruption accompanied by unprecedented growth of population as well as general economic inequality of medieval England. Reduction in yields and the emerging pandemic pushed the poor into the cities, cementing the urbanization trend and further worsening the epidemiological situation. The feedback loop erased a wide availability of labour for food production in rural areas, resulting in combined massive loss of lives and later stabilisation of population at the end of the pandemic.

Generally, the process of the feedback loop enhances pre-existing threats to the well being of the population in an increasingly hasty manor up to the point of major population numbers reduction [111]. Finally, as truly global food security disruptions occur relatively rarely, the uneven distribution of goods and lack of reliable logistical networks compliment the regional cases of the phenomena.

### Effects of the current coronavirus crisis on food security

#### Effects on modern agriculture and food production

The restrictions on movement and the need for physical distancing to keep people safe, along with requirements for additional personal protective equipment, reduce efficiency of various enterprises. While some industries adapted to the changing environment rather easily, space and cost reliant logistics as well as large scale production plants suffered during the pandemic the most. Likewise, movement restrictions and illness of employees are resulting in labour shortages [65] or even the closing of facilities. The meat industry became a primary COVID-19 hotspot in Europe due to reasons described by Nack [70] who also reported the numbers of infections among employees as well as closed meat plants. The dairy industry was mainly affected via reduced consumer demand for dairy products (especially in the hospitality sector) which resulted in a situation where milk even went uncollected from some farms [10]. Closing of the plants and reduced products sales from farms generally disrupted supply routes. In the US, the chain which supplies the food-service industry was forced to close and left an entire supply chain in limbo. That is especially true for five food staples such as beef, milk, eggs, and potatoes, which illustrate how the food system became a victim of its efficiency [43]. Given examples documented the sensitivity of modern agriculture with complex networks among farms, food industry, retail and hospitality sector to pandemics like COVID-19 in association with disruption of these complicated relationships.

One of the main features of modern agriculture is its absolute dependence on technology and industry support, such as mineral fertilizers [77]. The development of agricultural technologies over the last 150 years was accelerated by growing industry and enabled a significant reduction of human labour when the population employed in agriculture is continuously decreasing [11]. Authors documented that less than 2% of the population is directly employed in the agricultural sector of rich countries such as the USA or some European countries. On the other hand, this value can reach up to 70% in developing countries [11]. In contrast to this general global trend, the need for human labour varied among agricultural sectors and is traditionally the highest in fruit, vegetable, and horticultural speciality farms most often associated with the seasonal hand-harvest job [75].

Problems of modern agriculture in face of COVID-19 could be associated with a highly concentrated processing of agricultural products in the food industry. It is estimated that only about eight percent of farms in the U.S. supply food locally [46]. The rest feed a complex network that ensures restaurants and grocery stores across the country have a steady supply of hundreds of different products [43]. Strict hygiene rules already govern the production of food and there is no evidence that food poses a risk to public health with COVID-19 [28].

Country lock-downs and border closures tend to strongly impact farmers’ access to inputs like seeds, fertilizers, and agrochemicals [108]. In the People’s Republic of China, the production of pesticides declined sharply and only resumed gradually after production plants were shut down following the outbreak [74]. In West Africa, about 80–85% of smallholder farmers are at risk of losing all their dry season investments as a result of the lockdown due to COVID-19. More worryingly, there are almost no extension services except for the skeletal visit-and-train system. Farmers and processors are left without field demonstrations. They are unable to apply the critical second-phase urea fertilizers and appropriate pesticides [87]. Disruptions are also observed in various supply chains of farm equipment. According to CEMA [12], the issue with machinery components supplied by China has become a larger issue including European and North American supplies when manufacturers miss components that should have been produced in these countries because of ongoing closures or severe workforce containment measures. Amidst the growing pandemic uncertainty, public support for British food and farming has reached a record high as a result of farmers’ efforts to keep the nation fed throughout the coronavirus pandemic [73].

Since the outbreak of COVID-19, the agricultural sectors in many countries have been facing labour shortages, particularly those characterized by periods of peak seasonal labour demand or labour-intensive production. This is caused by limits on the mobility of people across borders, and lockdowns [74]. At the beginning of spring 2020 in Europe, farms rushed to find enough workers to pick strawberries and asparagus, later, border closures prevented the usual flow of foreign labourers. France called on its citizens to help offset an estimated shortfall of 200,000 workers [88]. Labour shortages have led to the rot of crops in farms.

### Effects on livestock and fisheries production

The pandemic is impacting on livestock sector due to reduced access to animal feed and slaughterhouses’ diminished capacity (due to logistical constraints and labour shortages) similar to what happened in China [32]. The effect of poor market access has been exacerbated by lower consumer demand, which has seen prices fall. U.S. pork prices, for example, dropped about 27% in just over a week in April [65]. There is a possibility of a disproportionately larger decline in animal protein consumption (as a result of fears—not science-based – that animals might be hosts of the virus, and other higher-value products like fish, fruits, and vegetables (which are likely to cause price slumps). These fears can be particularly true for raw fish products supplied to restaurants and hotels, including small and medium enterprises [65].

### Effect on food processing

The COVID-19 pandemic has led to reduced processing capacity following staff reductions due to lockdown measures; constraining meat and dairy processing industries, given their labour-intensive nature. In France, staff shortages due to childcare, quarantine, and sick leave have reached 30 percent in some slaughterhouses. There are similar instances in Egypt, Jordan, and Tunisia [33]. Food storage and conservation were further comprised. Transport disruptions and changes in retailing and consumption habits are forcing some collectors and processors to stock up. The pandemic also disrupted constrained informal businesses like meat and dairy processing in developing countries (i.e. up to 90 percent of volume). This disruption has mainly removed an outlet for small-scale producers, who cannot often sell to formal markets.

### Effects on food reserves

Food reserves are understood to be stocks of food held by a public entity on the local, regional, national, or international level. The food products should be of a nature to satisfy caloric and/or nutritional requirement of a given country population. In most cases, food reserves are built up from grains or other staple foods. There are various uses for food reserves, amongst which management of food crises stands in the foreground in light of the current COVID-19 pandemic [29]. The size of food reserves varies by country. Generally, it is assumed that a person consumes between 160 and 175 kg of grains per year and the reserves should be worth approximately three months of consumption. Similarly, some countries adopted an approach to creating reserves that would be equal to three months of market demand [30].

While the size of food reserves is not communicated to the public in most countries, the following Table 1 shows the size of food reserves for several selected developing countries based on [30, 52] and European [29].

**Table 1:** Size of food reserves for selected developing countries. (via [29, 30, 52])

Country	Grain Reserves (tons)	Population	Grain reserves per capita (kg)
Zimbabwe	936,000	14,440,000	65
Kenya	720,000	51,390,000	14
Malawi	180,000	18,140,000	10
Bangladesh	1,500,000	161,400,000	9
Ethiopia	205,000	109,200,000	2
Nigeria	325,000	195,900,000	2

Table 1 shows various levels of food reserves that correspond to countries’ goals, abilities, and perception of risk. The table reveals some salient points. Zimbabwe with the lowest population has the highest per capita grain reserves, unlike Nigeria. The grain reserves per capita give an estimate of the amount of grain available to an individual in a country at a given time. Bangladesh is also an important case with 1.5million tons of grains one which is reasonable, however, its seemingly high population has reduced the amount of these grains available to an individual in the country. According to FAO [30], the quantity of food in reserves should be approximately three months of consumption as a person should consume between 40–43.75 kg of grains within this period. A closer observation of Table 1 shows that only Zimbabwe meets this criterion. This illustration paints a picture of the global food crisis. With the ongoing pandemic and initial interventions of governments at the beginning of the COVID-19 pandemic in the food commodity markets to increase the stockpiles, it can be assumed that the food reserves may rise to new high levels. In contrast, food reserves may fall drastically especially in low-middle income countries where these reserves are meted out as palliatives. Also, the farming season was hindered by COVID-19 lockdown and restrictions and it is predicted that harvest will barely serve for immediate consumption and little or none for reserves.

Whereas in the CZ approx. 50% of the total silage maize production (2.474 million tons of dry matter according to the Czech Statistical Office—CZSO) represents a substrate for numerous agricultural biogas plants (BGPs). This means the area of approx. 5% of the total arable land. On the other side, the cattle as a major farm livestock consumer of maize consumes approx. 40% of the total production. The remaining 10% falls into storage losses and seeds. A recent study by Pulkrabek et al. [84] showed a comparison of four Czech regions (areas on the level of NUTS 3) where the production of silage maize is insufficient in some of the regions with traditional dairy cattle production accompanied by a higher number of BGPs. They depicted the Vysočina region, as one with an insufficient production-consumption ratio. This region with 273 027 ha of arable land (11% of the total arable land in CZ) every year lacked approx. 65 thousand tons (approx. 10%) of dry matter silage maize to meet their needs. The gap between production and consumption in the overstrained regions, such as Vysočina, is normally compensated by other regions. But in the case of maize, there are no reserves and the share of arable land producing silage maize already reached its maximum due to the growing restriction on erosion-endangered soils. Therefore, even the slight decrease in the yield of about 5 -10% in the total production would result in the overall scarcity of feedstock either for dairy cattle or for BGS [84]. On the other side, such a model shows the important fact that by using various measures Czech agriculture still has a production potential to cover the needs of the population and livestock even in case of a severe yield decline say in the advent of the scarcity of mineral fertilizers. One of such measures could be the cutting of the least emerging consumer, which is the energy production from purposely grown agricultural biomass. Since its share of the total power energy production is approx. 3%, such a source could be expendable in the case of crisis and the fallow land could be used for the production of silage maize for cattle. This way even in case of a 50% or lower decline in the silage maize compared to the average decennial yield of 12.8 tons per ha of dry matter (CZSO, 2020) could fully meet the needs of the current dairy cattle population.

### The potential for a change

Historically, pandemics were associated with infectious diseases. They devastated large areas of Europe. In the same way, they impacted the population of other continents. However, modern society changed this picture. Nowadays, non-infectious diseases account for the vast number of deaths. For instance, the server worldometers.info indicates that, in 2020, up until July 8th, the number of deaths caused by cancer amounted to 4,268,200 and the number of deaths caused by communicable infectious diseases (such as cholera, influenza, hepatitis, malaria, measles, or tuberculosis) amounted 6,746,700. The server does not point out cardiovascular diseases (CVD) of non-infectious origin. Using WHO estimations [110], CVDs are supposed to account for the death of 17.9 million people a year (it is about 8 million people in half-year period as reported for cancer of communicable infectious diseases earlier by worldometers.info). These data document that non-infectious diseases (moreover, not all such diseases are included in the data presented here) account for about 2 times higher number of deaths than infectious diseases. Giddnes [44] writes that about 70 percent of death in Western countries are attributable to four types of illnesses mostly of non-infectious nature (cancer, heart disease, strokes, and lung disease) and they are related to the lifestyle of various type of social groups. The media even wrote about civilization pandemics when referring to non-infectious diseases. However, the outbreak of the COVID-19 disease created a different situation. This infection disease overshadowed the non-infectious (and dominating) ones, although people with non-infection diseases are the most vulnerable to the SARS-CoV-2 coronavirus infection [14]. Non-infectious diseases such as CVDs are associated with poor diets and inappropriate eating habits existing in some social groups but COVID-19 shows that this is also the case of infectious diseases [3].

The agenda within the first quarter of 2020 was ruled by SARS-CoV-2 coronavirus and related COVID-19 disease (the number of the deaths in years 2020 and 2021, amounted to 14.9 million according to who.int). While the reason for a change of interest from non-infectious diseases to the infectious ones despite the remaining majority in cause of deaths remains unclear, we argue that the shift is because of the change in risk perception. While in the last decades (i.e. from the 1980s) the discourse was preoccupied with understanding the risks as depending on human decisions and being industrially produced [5], COVID-19 strongly indicated again that risks are of natural origin as well. We easily forget the lessons of the past when risks were attributed to nature or God. Contemporary modern world (or using Bauman late modern world) and especially its industrialized (developed) countries have not been exposed to extreme natural disasters blocking the operation of whole continents (enormous earthquakes, serious global infectious pandemics, large volcano eruptions) for a long time. That is why the shift towards man-made risks started to be accentuated during the last decades. Not only nuclear disasters of Chernobyl type but also climatic change impacts are not only of natural origin. To a large extent, they are attributed to human activities. That is why the risk was in recent discourse considered to be unexpected outcomes of human activities instead of being also attributed to hidden meanings of nature, as it was in the past [45].

Since COVID-19 is a risk of nature-related background (we refuse conspiracy theories about the artificial origin of this infection because it would bring us back to the modern understanding of risks – as man-made) we think the COVID-19 situation described as a crisis opens the opportunity window for novelties. Using the ideas of reflexivity, COVID-19 has also opened the window to reflect on our past. We might learn, for instance, how such risks (“nature made”) have impacted society in the past (compared to contemporary man-made risks). Can we mobilize the past [112] to learn from the past reflexively and mitigate the impacts of such a natural-based pandemic as COVID-19?

COVID-19 destroyed the normality (the practices and systems taken for granted) and as such changed everything. These changes led to a period of crises [57]. It makes the situation riskier because our experienced practises and systems are eroded. Such a situation is the best incubation time for novelties [112]. COVID-19 exposed the vulnerability of our agri-food systems (slaughterhouses were the most prominent hotspots of COVID-19 and in many countries, the workers were either migrants or from lower-class and ethnic groups more exposed to COVID-19).

Some reflections on the risks of COVID-19 and the crisis produced by this disease are already on the agenda concerning agriculture and food. Various stakeholders (academicians, farmers, activists) from various countries reflected on Agriculture and Human Values (AHV) journal their thoughts about COVID-19 and its impacts on agriculture and the food industry. The general idea underpinning all reports on COVID-19 should be seen as the opportunity window for transitions in agriculture and food sectors towards sustainability. As Dranhofer [17] notes, “Yet if there is one thing that the current COVID-19 pandemic has shown, it is that much of what was unthinkable may suddenly become a reality”. The situation in the crisis enables unbelievable solutions to minimise the risks. The authors in AHV unanimously argue for sustainable food systems emphasising biodiversity, resilience, renewability. Such systems are juxtaposed to global agri-food chains considered to generate food injustice [22, 47]. In their view, it is the lesson learned from the COVID-19 crisis and how to deal with future risks that might come suddenly and unexpectedly [83]. Some of the authors [72] reflected on migrant workers (or workers in general) in the agriculture and food sector showing paradoxes (e.g. paradox of closed borders for international travels and bringing the migrant workers to countries of Global Norths /Germany/ from COVID-19 hotspot regions of Balkan) and importance of migrant workers for contemporary agri-food regime. Others [3] highlighted the links between diets and types of social groups (ethnics social groups, poor people and their vulnerability to pandemic due to inappropriate patriate diet). The new pathway [17] is seen also in utilizing ICT in agriculture (mostly for communication and marketing) [39]. Some of the authors [7, 49, 66] in AHV echoed the idea of human-nature balance since they consider COVID-19 as the expression of environmental problems (these problems enable easier animal-human infections transfers, for instance). In countries of Global South [37, 69], the issue of COVID-19 related to the questions of food security (interestingly, it was not the case for Global North apart from a discussion on food sovereignty).

We are the successors of those who survived significant climatic changes, periods of famines, and pandemics. This is attributed to the evolution of mankind being a complicated process demonstrating a trade-off between the benefits of our progress and myriads of victims among our forebears.

Hunger and diseases have been cohabitating with us since the beginning of humankind. Their impacts were more intensified when agricultural societies emerged, and the human population expanded. The changes from hunting and gathering or pasturing to agriculture are considered to be beneficial for humankind when such transition was completed. During a relatively short period, a reliable source of food was at disposal and new methods of storage increased the probability of survival, mostly in the severe winters of today’s central Europe [92]. People settled, changed their lifestyles, natality grew, and mortality decreased.

Wars, an endeavour of some societies to conquest new territories and resources or to spread their cultures resulted in deaths, hunger, and epidemics. As human settlements evolved these impacts were more often and cruel. Hunger was related also to the impacts of climatic changes. Even local and relatively short-time change of the weather was able to decrease yields to such a level that the local population was exposed to famine. As agriculture evolved and with bettering farming procedures, the plants achieved the limits of their natural possibilities in the regions facing the threat of drought, frost, or wet seasons [92].

Under such a situation, only one year with significant weather divergence from normality was enough to start the series of events resulting in hunger. The water reservoirs were depleted, herds were slaughtered and finally many farms were abandoned. Pauperized families (if they survived) migrated to cities with the hope to find some subsistence. It resulted in a growing number of urban poor. It is the reason why cities were overcrowded before the crisis which facilitated the spread of epidemics.

### The inverted case?

While the COVID-19 pandemic emerged without a prior food security crisis, the link between the two phenomena might not be inverted. With the effects of globalisation, the dynamics of a spread of respiratory diseases changed dramatically [76], especially due to the speed of air travel. The resulting disruptions on logistics and production capabilities do pose a real threat to the food security of the poorest, yet the extend remained far from famine. Even though the scope of current observation is certainly not complete, the existing threats seem limited on the global scale, as the aforementioned disruption of logistics had a relatively short span of existence [53]. Outside of the realm of logistics, COVID-19 pandemic threatened food security via the lack of available workforce and illness driven reduction of production capabilities. Besides the production an unprecedented element of food reserves allowed for a regional stability in case of minimal imports.

The resulting comparison of the empirical data and the contemporary events questions the validity of the established link between food insecurity and pandemics. Despite the inversion of core phenomenon, the main elements of the link remain interconnected in a strikingly similar manor. The connection between economic inequality, nutrition and vulnerability to infectious diseases remains valid. Disruption of food production in a time of a crisis poses an everlasting threat to the stability of supply. And finally, the issues of limited production imprint on the motivation of those who leave rural areas, resulting in more urban poor further feeding into more infectious locations.

In light of the comparable elements, the outlook suggests a validation of the link between pandemics and food insecurity. Albeit the link can in terms of dynamic progress of humanity alter its elements as well as the resulting function of the feedback loop, the core aspects of the connection remain reliably interconnected. For example in the case of CZ, despite being relatively mild, the threats to food security of the population and especially its poorest parts remains a valid cause for concern.

### Alternative food resources during pandemic and food shortage

From Central Europe, there are available data on famines from chronicles since the tenth century, and sometimes they indicate how the population dealt with the situation. In the following text, we will therefore focus on the solutions, specifically on alternative food sources.

The basic source of food was grain. The risk of crop failure had been reduced by growing other crops, especially legumes or buckwheat. An extremely important chapter was the introduction of potato cultivation in Europe. The population was initially distrustful of the cultivation of this crop, and so potatoes began to be grown to a greater extent, despite repeated recommendations and regulations of the authorities, first after the famine in Ireland in the mid-seventeenth century and Central Europe only after a catastrophic grain crop failure in 1770 [60, 82].

Although it was possible to consume the whole grain (porridge, groats, “pražmo”- roasted unripe grain), it was predominantly milled into flour, from which bread was mainly made. There was a demand for other means to set flour with less valuable components, or which alternative cereals and pseudo-cereals, even from the ranks of wild plant species to use [8, 98]. Acorns were commonly used, from which relatively high-quality flour can be prepared (e.g. in the humid years of 1678 and 1771, when there was a grain crop failure, [98, 99]. Beech acorns (famine of 1571) or even horse chestnut acorns [24, 58] are also mentioned as an important source of emergency food. Substitute flour was ground from rhizomes of quack-grass (*Elytrigia repens*) or clover heads [23, 58]. In the shortage of grain, the dough for baking bread was set with turnips (*Brassica campestris rapifera*) or later also potatoes, but also lichens and peat moss [58, 113].

Flour from seeds of wild and weedy species of grasses was also used for making bread. This applies in particular to the species fingergrass (*Digitaria sanguinalis*), foxtail millet (*Setaria italica*), and occasionally wood millet (*Milium effusum*), the first two of which were also intentionally grown in the past. Furthermore, alternative pseudo-cereals were rhizomes of cattail (*Typha* sp. div.), seeds of amaranth (*Amaranthus* sp.), goosefoot (*Chenopodium* sp. div.), saltbush (*Atriplex* sp. div.), and some wild legumes such as meadow pea (*Lathyrus pratensis*) or black locust (*Robinia pseudacacia*) [24].

The problem was supplementing the flour with low-value or even completely harmful components, such as hay, straw, bran or finely-ground wood, tree bark (especially from birch), and pinecones. In the sixteenth-eighteenth century, reports of the miraculous finding of flour in the field and thus of baking bread from marl were repeatedly recorded in various Central European regions [98]. It was the lower quality of food that further exacerbated the effects of the famines. Stinking meat from various carcasses, for example, was also eaten. As a result, various indigestion and diseases were common, which made the situation even worse. In extreme cases, cannibalism occurred (in the Czech lands, specifically in 1028, 1281/1282; 1312–1315).

Recommendations for the use of alternative food sources in the starving years appeared more frequently in Central European literature during the eighteenth century. However, the publication of advice and manuals on what to eat in times of need increased significantly as a result of the year without the summer of 1816, with the most important manual in Bohemia being written by Matyáš Kalina von Jätenstein [58]. Their publication continued during the nineteenth century until the twentieth century when they responded to a period of scarcity during World War I. The advice varied in quality and could sometimes have resulted in a further deterioration in the health of the population; Karel Domin [23, 24] already had scepticism about them. Crisis manuals are still published today—most recently in the CZ, in response to the coronavirus crisis, a cookbook by Eva Francová [38] was published.

A specific option for obtaining food is hunting wild animals. It does not seem to have been more important in Central Europe in the past. The right to hunt belonged only to the nobility and poaching was intensely persecuted and severely punished. Also, the number of games was probably not very high. However, the current connection between hunting and the COVID-19 pandemic is interesting. Many regions in Europe and North America have long-term difficulties with game overpopulation and related damage to ecosystems [4, 20]. Furthermore, wildlife hunting here stagnated due to the COVID-19 pandemic, which was caused by the deteriorating sale of games due to the closure of restaurants and some processing plants [64]. The situation is quite different in poor regions, such as Africa, where the economic impact of the COVID-19 pandemic has led to an increase in poaching. Due to higher food prices, reduced job opportunities, and the collapse of tourism income, some people living in and around national parks have had to turn to the forest to survive, including hunting wildlife for meat. This also applies to critically endangered animal species, such as mountain gorillas (*Gorilla beringei beringei*) or black rhinoceroses (*Diceros bicornis*) [48, 91, 96].

The question is in what direction humanity will adapt to possible future famines. Further crop breeding is certainly needed to increase yields and improve resistance to the adverse effects of the environment, in which genetic modification will undoubtedly play an important role [85]. In vitro food growing can also be essential—from plant foods, especially algae, or from animal tissue culture [6, 18].

### Strategies to ensure food access and self-sufficiency during a pandemic

Food security can be ensured either by food self-sufficiency (food production in a territory from own agri-food resources), by food import, or by a combination of both, which is the current situation in most countries of the world [15, 39].

Food self-sufficiency can be defined as the degree to which a country can meet the need (demand) for food from its resources [31]. The degree of food self-sufficiency can be quantified either using the calories framework or using the monetary framework. The degree of self-sufficiency can be expressed not only in general but also for the main agricultural products or food groups [15]. Although food self-sufficiency is at odds with the globalization trends of recent decades, recent crises show the need to adopt a strategy to ensure food security. Whether self-sufficiency can be a feasible strategy has been discussed by many scholars [15, 42, 59].

Clapp [15] discusses food self-sufficiency in light of the 2007–2008 international food crisis that was caused by increasing food commodity prices and food commodity prices volatility. The article discusses and broadens terms of food self-sufficiency whereas argues that rather than having contrasting food self-sufficiency versus international trade, it is important to seek a certain middle-ground. This means that rather than outright rejection of international trade, it is important to assure that individual countries’ capacity for food production should be enhanced. Using this approach, it would be beneficial according to Clapp [15] to create food self-sufficiency policies that would be compatible with the international trade rules of WTO by increasing their flexibility [15]. These contrasting ideas were evident through the actions of individual countries during the recent COVID-19 crisis where some countries were shutting borders and hence effectively prohibited exports (Thai rice), while some countries advocated the continuance of opened borders and assurance of international trade even at the peak of COVID-19 crisis.

The issue of food self-sufficiency has also been discussed in light of recent COVID-19 pandemics [19, 34, 107]. For example, Sers and Mughal [34] discuss the issue of self-sufficiency of West-Africa in rice during the recent COVID-19 outbreak and preventive lockdowns that led to the stretching of food supply chains and subsequent diminishing of production and transportation capacities. Albeit increasing self-sufficiency, still, about 30 percent of rice consumed in West Africa is imported. According to the authors, this was also reflected in the global food commodity prices that increased by over 50% from the beginning of 2020 to April 2020. This led to increased vulnerability for the African population in terms of food security. To solve this problem, the authors suggest among other solutions enhancement of agriculture financing that could improve farmers’ access to innovation [34]. This may also be the case in the CZ where possible reliance on migrant workers for particularly fruit and vegetable production can be solved by technological innovations in the field of automation and robotics. In contrast, this also showed the dynamism in the effects of COVID-19, in a location lack of technology was a problem in another it was absolute dependence (see Effects on Modern Agriculture and Food Production Section). Woertz [107] discusses food security in Gulf Arab countries and the impact of COVID-19 on food availability/accessibility in the region. The author also mentions the failures of food self-sufficiency policies in the region due to increasing water scarcity. Foreign farmland investment is another interesting policy mentioned by the author, who mentions that this policy failed due to various commercial, political, and socio-economic factors. Woertz [107] argues that Gulf Arab countries have accepted that food imports are necessary, and it is important to better manage entire value chains. For example, he suggests that countries must enhance food storage capacities to bridge supply shortfalls. At the same time, there is a need to better protect vulnerable parts of Gulf Arab countries’ societies such as migrant labourers. Woertz asserts that poor people of Gulf Arab countries were more threatened by COVID-19 due to the nature of their jobs and living arrangements. At the same time, obesity prevalence in the analysed countries can also negatively influence the ability of people to cope with the COVID-19 pandemic. Generally speaking, according to Woertz, agri-food systems in the Gulf Arab countries performed relatively well with little disruption due to COVID-19. This is akin to the situation in the CZ where no major disruptions in the agri-food systems occurred during the pandemic. Deaton and Deaton [19] examined the effects of COVID-19 pandemic on Canadian food security. While temporal demand surges and disruptions in supply were observed, according to the authors, no significant price appreciation was recorded. This means according to the authors that there was adequate food supply for Canada in the observed period and no major issue related to food security appeared. The authors, nevertheless, suggest three major areas of interest to be examined to ensure food security in the future. These are ease of capital flows (achieved through a lowering of interest rates), international exchange (achieved through opened borders), and assurance of transportation (remote locations of Canada). Similar to the CZ, temporary foreign labour also plays a dominant role in fruit and vegetable production in Canada. Due to possible border closure, parts of Canadian agriculture could be threatened by a lack of labour. From the consumers’ perspective, it is reasonable to expect minor alterations in consumers’ baskets as a reaction to the temporary unavailability of certain food items. This happened similarly in the CZ where temporary spikes in demand for certain goods existed (e.g., flour for home bread-making). The authors point to the necessary existence of international exchange which non-existent may primarily threaten the most food-insecure countries. While claiming that domestic transportation in Canada may be vulnerable to large distances among provinces, this issue is not a concern for smaller countries such as the CZ.

The second strategy on how to ensure food security is through food imports. Food imports must be adjusted for possible re-exports of food. A typical example is a milk that is re-exported from Germany to the CZ [103]. Kinnunen et al. [59] measure and calculate the minimum distance between the source of food production and final consumption for six crop types that are prevalent around the globe. Researchers determined that less than one-third of the global population can satisfy their demand for specific crops within a radius of less than 100 km. Specifically, this varies based on different crops and regions between 11 and 28 percent. Furthermore, for more than one-quarter of the global population, this distance is more than 1000 km. Specifically, depending on region and crop, the estimated share of the population is between 26 and 64 percent [59]. For instance, for most parts of Europe and North America, wheat as one of the most important temperate climate crops can be obtained within a radius of 500 km. This is in harsh contrast to the global average radius of almost 4000 km. This example clearly shows that current systems and technologies of food production together with consumption patterns are at present not coherent with the idea of local production and consumption. It is important to note that any increase in locally produced and consumed food may lead to issues such as water pollution, increasing water scarcity locally, and vulnerabilities during crises caused for example by poor harvests or mass migrations. This can also be the case of potential vulnerabilities—food crises related to border shutdown as recently experienced with COVID-19 [1]. Gerten et al. [42] discuss food security in terms of four inter-linked global boundaries (biosphere integrity, land system change, freshwater use, and nitrogen flows). They argued that transformation towards more sustainable production and consumption patterns may provide enough food resources for more than ten billion people. This also depends on the proper spatial re-distribution of cropland so that more local food production is assured [42].

## Conclusion

In light of the lessons learned from the history, the contemporary COVID-19 pandemic caused multitude of troubles on the global scale. While it altered from most previously recorded cases of pandemics in terms of its link with food security, the core aspects of the connection manifested across both society and economy. The inversion of the historically established sequence of events might have been partially responsible for a relatively limited loss of lives as well as for establishment of a precedent for future strategies for fighting such crises. Even though the reason for the inversion remains insufficiently explored, the general outlook points towards globalisation and its effects on the distribution of goods and knowledge.

Labour shortage was one striking consequence predominant during the crisis as it affected all elements of the food value chain. Slaughterhouses were prominent hotspots for COVID-19 which revealed the vulnerability of the agri-food system. Furthermore, fruits, vegetables and horticultural speciality farms are traditionally known for manual harvesting was significantly affected. International relations were the major cause of labour shortage and also an underlying factor in agri-food sustainability as the existing lacuna between food security and food sovereignty widens.

Apart from hunting as an alternative food source during a pandemic, further crop breeding is certainly needed to increase yields and improve resistance to the adverse effects of the environment, in which genetic modification will undoubtedly play an important role. In vitro food growing can also be essential—from plant foods, especially algae, or from animal tissue culture. Food security can be ensured either by food self-sufficiency, food importation, or a combination of both. Instances, about schemes implemented by the governments were highlighted.

The revelations from this study are expected to instigate the reformulation of government policies, upgrading of food reserves and, the explosion of alternative food sources towards the systemic development of global food systems as an interconnected network. The argument about the complacence of mankind due to less occurrence of natural disasters and warlike events compared to the era during and before the twentieth century is a question that begs an answer. Hence, this may be another underlying impediment to the advancement of food security.

## Data Availability

Not applicable.

## Abbreviations

AHV: Agriculture and Human Values
BGP: Biogas plant
COVID-19: Coronavirus disease 2019
CZ: Czech Republic
CZSO: Czech Statistical Office
FAO: Food and Agriculture Organization
ha: Hectare
HIV/AIDS: Human immunodeficiency virus / acquired immunodeficiency syndrome
IATP: Institute for Agriculture and Trade Policy
ICT: Information and communication technologies
km: Kilometre
NUTS: Nomenclature of Territorial Units for Statistics
SARS-CoV-2: Severe acute respiratory syndrome coronavirus 2
U.S.: United States
WHO: World Health Organization
WTO: World Trade Organization
